# Nurse Marion's Romance

**Published:** 1887-04-23

**Authors:** Kate Furley


### Nurse Marion's Romance.

By Kate Furley.

Chapter V.—Complications.

Marion would have faced any unknown danger at that moment rather than meet the familiar countenance of Dr. Brandreth. She felt that she must defend herself, but could invent no plea that she could put before this man who was both her lover and her official superior. In the one capacity she had incurred his anger by disobeying one of the most obvious and inevitable rules of conduct for a nurse, and in the other she had wounded him more deeply than she cared to think of. Only one reply to his impending charge could have justified her—that she was to become Holtayne's wife—and this it was not yet in her power to plead. It was indeed one of the things which "go without saying"; she did not doubt that such was to be the end of this unexpected renewal of old relations, and doubtless Jack assumed that she understood this without his stating it directly; still it is one of those things which a woman likes to have distinctly said, and which, however firm her conviction may be, she cannot possibly repeat till it has been put into words by the man who seeks to marry her. And although she believed that all would end well, and that her happiness would not be materially affected by the accident of that day, she was annoyed at having, in a moment of weakness, however excusable, tarnished a hitherto stainless record by incurring the risk of dismissal under circumstances which could not, from a hospital point of view, be regarded as other than humiliating.

Her face looked very indignant as she entered the little room where the doctor was waiting for her, and he could not guess that the indignation was directed against herself; nor could she know how little of personal anger there was in the feeling that contracted his brows and made his lips keep so firmly closed.

For a moment they stood facing each other from opposite sides of the apartment. Then Marion said: "You sent for me, Dr. Brandreth. What is it you have to say to me?" and set her face to hear the worst.

"I wished to say," he answered slowly, "that I am sorry I spoke to you so angrily as I did just now." The words did not come easily, for he was proud and naturally reticent; but he had an almost fastidious sense of justice, which made him realise within a moment of his uttering it, that his rebuke to the nurse had been only a vehicle for his own irritation.

"What you said was perfectly just," answered Marion. "I was in the wrong, and you were within your right in finding fault with me."

"Within my right—yes; but you know as well as I that it was not the mere trifling fact of your cap not being on your head that made me speak as I did, and I feel that I owe you an apology for making a pretext of such a trivial cause to give vent to an anger which, I suppose, I have no right to feel."

"No apology was necessary," said Marion, coldly, leaving unanswered the suggestion conveyed in his last words.

There was silence for a moment, but each felt that the interview was not yet at an end. After a few moments Marion asked, "Have you anything further to say?"

Dr. Brandreth hesitated. "Yes," he answered, speaking slowly and with apparent difficulty. "Yes, I wish to say something for which I must ask your pardon in advance."

"You are unnecessarily humble, surely," returned Marion, in a tone of suppressed irritation.

A slight frown on the doctor's face showed that he felt the implied scorn, but he spoke in a studiously quiet voice:

"It is doubly difficult for me to make any suggestion to you just now with regard to your conduct, because I fear that you will misunderstand my motives for doing so. It is almost inevitable that you should; and I have already confessed that only a few minutes ago I took the opportunity of your being guilty of a fault, to display an anger which, I admit, arose from another cause. Yet I ask you to believe that in what I am going to say, I speak not as a disappointed lover, but as a friend."

"If I have neglected my duty as a nurse, you have, of course, the right as physician in attendance on my patient to find fault with me."

"Unfortunately my—advice—is addressed to you less as a nurse than as a woman."

"That is a point which—excuse me—does not concern you."

"I might reply with justice that it does. You must be as well aware as I, that the quality which has made you specially remarkable as a nurse is something apart from the skill which mere training can give. You have given the patients under your care a sympathy and womanly tenderness which are quite as important as due attention to physical needs. You have often helped recovery, and made my work easier by no other means."

"I think you over-rate my powers," said Marion, replying with coldness to the doctor's earnest tones.

"I think not; but, at any rate, you must admit that I am only telling you what you are said to be, and I do not wish you to act unworthily of your reputation."

"And am I doing so?"

"In one sense you are. You are better known than you think. I have sent several patients to the farm here to complete their recovery. I use it almost as a convalescent home. Some of these came from St. Lazarus', and they have spoken of Nurse Marion so much, and in such terms of praise and respect, that the woman of the house had grown, without knowing you, to think you something of an angel."

"And she finds, now she does know me," supplemented Marion, "that I am not angelic, but exceedingly human. I am sorry to disappoint her expectations, but I fear I cannot live up to them. It is unfortunate, but inevitable."

"Why do you insist on taking amiss everything that I say?" cried the doctor. "You give to my ideas a colouring they were never meant to take, and do your best to put me in a false position. Be just, Marion.

Admit that, now at least, I am speaking in no selfish spirit. I claim no rights over you: I ask no questions —not even whether this old lover of yours whom chance has brought to your feet once more is in any way worthy of you. But is it too much to wish that you whom I have regarded as an ideal woman, should not stand lower in the eyes of others than you do in mine?"

"I do not know. I am indifferent to the opinion of the world."

"You are wrong to be so indifferent. Remember that no one but myself knows the truth about you and Holtayne, and that to the eyes of others you are—if I must state the fact plainly—presenting the undignified spectacle of a nurse flirting with a patient."

"Dr. Brandreth, how dare you speak so to me!"

"Because of my regard for you, but not because I have asked you to be my wife, and you have refused. I honour you too much to be willing that any aspersion should be cast on your behaviour."

"I do not see how you can prevent that better than I."

"I think I can. It is easier for me to mention casually that you were once engaged to Mr. Holtayne, than it is for you to volunteer such a statement. I can explain more fully than you could, without seeming to boast of your devotion to duty, the circumstances under which your engagement was broken off. May I do so?"

"I do not see why things affecting my private life should be told to everyone."

"Your position here is public; your actions are public, and require public explanation. As physician of your ward, I have a certain prescriptive right to be looked upon as your friend and protector, and can authoritatively state the facts that justify your present attitude. Believe me, I have no wish to gratify mere curiosity; but I certainly desire—can you blame me for it?—that you should continue to stand as high in all eyes as you do in mine."

He spoke so earnestly,—his abandonment of his former pretensions to her favour was so complete and unselfish, that he broke the mask of obstinacy she had worn, though her answer still took a petulant tone. "Why do you take so much trouble about me?" she exclaimed.

Dr. Brandreth did not reply to her question; but assumed her consent to his request. "Then I shall take the opportunity to make things clear. I had better add that you mean to marry Mr. Holtayne on his recovery."

"No, you must not do that."

"Why not?"

"I had rather you did not!"

"It would silence talk more effectively than any other statement."

"I forbid you to say it," cried Marion. "If it is necessary to say anything of the sort I will tell it myself."

"I think you must admit the necessity. If I may tell half the truth, why should I not tell the whole?" asked the doctor.

"You are under a false impression. There is at present no engagement between Mr. Holtayne and me."

"Marion!" exclaimed Dr. Brandreth in a voice in which surprise contended against gladness.

"At least"—she altered her phrase—"no definite engagement."

"What am I to understand by that?"

"Is it necessary that you should understand anything?"

"This certainly puts a different aspect on the matter. I will not deny that the fact that you are still free makes me more hopeful," he said.

"I am not free," she answered; "that is—there are things which do not need to be put in words."

The doctor smiled dubiously. "I am dull-witted enough," he replied, "to think that all things that are worth saying should be said as clearly as possible, and in this case—to be frank with you—open speech is so desirable, that I should construe silence unfavourably."

The suggestion conveyed in his words vexed Marion, because it struck an unexpected echo in her own mind. She had trusted Jack implicitly till within a moment of her coming to meet Dr. Brandreth; she had felt sure that she understood all that was in his heart; she had assumed that only his weakness had hitherto restrained the expression of remorse and re-awakening tenderness and long-delayed loyalty; yet now when she had been forced to analyse the relation in which they stood to each other she felt no assurance that he had any real affection for her. The unavowed doubt made her colder than ever to the doctor.

"You assume things too hastily," she said. "Everyone is not so direct in utterance as you."

"You are very determined to take my forlorn hope away from me," he answered.

"There is no question of hope," she said firmly. "My being bound or free will not affect you."

"I cannot admit that. The month which was to elapse before you gave your final decision is only half over; till it is past I will not despair. I promise, however, not to trouble you with my wishes till the next fortnight is past. Meanwhile have I your forgiveness for what I have said to-day?"

"Oh! certainly," she answered, with an appearance of indifference she did not feel. "I can't doubt that your intentions are kind, if your actions did seem a little officious. As you have nothing more to say to me I will go, as any patient may require me. After a scolding," she added sarcastically, "I must be doubly careful in the performance of my duties."

She was more than half ashamed of the tone in which she spoke to him, but the bitterness in her own soul was too keen to be wholly suppressed, and it seemed natural to let the expression of it fall on the hapless doctor.

She did not, however, return to Holtayne's room directly. She was irritated and annoyed by what had passed, and her nerves were more shaken than she liked to acknowledge. So she went out to the garden at the back of the farm-house, an ill-kept, old-fashioned place, where daffodils bloomed peacefully in the shadow of budding gooseberry bushes, without feeling any indignation at the chickweed sprouting cheerfully around. The beauty and the ugliness of it alike escaped Nurse Marion to-day; the only merit it possessed in her eyes was that it was so rarely entered by any of the inhabitants of the house, that she could walk up and down its weed-grown paths till the spring breeze had cooled the fever in her pulses without fear of interruption.

But even in this she was disappointed. Before many minutes had passed she saw the mistress of the house coming towards her with a letter in her hand.

"The doctor left this with me," said the farmer's wife. "He forgot to deliver it when he paid his visit to the gentleman."

Marion took the letter, but saw in a moment that it was not for her; it was addressed to Holtayne at the hospital of St. Lazarus.

"This is not mine," said she, giving it back. "Why have you not given it to Mr. Holtayne?"

"I thought you would take it to him yourself, nurse," answered the woman, with an unnecessary demureness which annoyed Marion.

"Give it to him at once," she said, in a sterner tone than she was accustomed to use, handing the letter to the other.

She had barely glanced at it, yet she had perceived that the envelope was of the newest fashion in shape and tint, that the address was written in a bold but ill-formed—one would say half-educated—feminine handwriting, and that on the seal was a swallow in full flight, with the motto "*Me Voici.*"

That flaunting "I am here" had a very irritating effect on Nurse Marion's nerves, already jangled by the events of the day, and her curiosity was the more easily aroused that this was the first letter Jack had received since he came to the hospital, and that the post-mark was London. What lady was there—Marion knew the writer was of her own sex—in the same city who had the right thus to herald her epistle with this *Me Voici!*

(*To be continued.*)

